# The fate of recent duplicated genes following a fourth-round whole genome duplication in a tetraploid fish, common carp (*Cyprinus carpio*)

**DOI:** 10.1038/srep08199

**Published:** 2015-02-03

**Authors:** Jiong-Tang Li, Guang-Yuan Hou, Xiang-Fei Kong, Chun-Yan Li, Jian-Ming Zeng, Heng-De Li, Gui-Bao Xiao, Xiao-Min Li, Xiao-Wen Sun

**Affiliations:** 1 CAFS Key Laboratory of Aquatic Genomics and Beijing Key Laboratory of Fishery Biotechnology, Centre for Applied Aquatic Genomics, Chinese Academy of Fishery Sciences, Beijing, 100141, China; 2 College of Fisheries and Life Science, Shanghai Ocean University, Shanghai, 201306, China; 3 Tianjin Fisheries Research Institute, Tianjin, 300221, China; 4 College of Life Science and Technology, Dalian Ocean University, Dalian, 116023, China

## Abstract

Whole genome duplication (WGD) results in extensive genetic redundancy. In plants and yeast, WGD is followed by rapid gene deletions and intense expression differentiation with slow functional divergence. However, the early evolution of the gene differentiation processes is poorly understood in vertebrates because almost all studied WGDs are extremely ancient, and the genomes have returned to a diploid status. Common carp had a very recent fourth round of WGD dated to 8 million years ago. It therefore constitutes an ideal model to study early-stage functional divergence and expression differentiation in vertebrates. We identified 1,757 pairs of recently duplicated genes (RDGs) originating from this specific WGD and found that most ancestral genes were retained in duplicate. Most RDGs were conserved and under selective pressure. Gene expression analysis across six tissues revealed that 92.5% of RDG pairs were co-expressed in at least one tissue and that the expression of nearly half pairs ceased to be strongly correlated, indicating slow spatial divergence but rapid expression dissociation. Functional comparison revealed that 25% of pairs had functional divergence, of which neo- and sub-functionalization were the main outcomes. Our analysis revealed slow gene loss but rapid and intense expression and function differentiation after WGD.

Whole genome duplication (WGD) is a dramatic event that results in the duplication of genome sequences. Although WGD leads to an increase in gene numbers, the subsequent diploidization process returns the genome to a diploid-like condition^1^. Studies have summarised at least three commonly accepted and different divergence functions of duplicated genes during the diploidization process: 1) one copy is suppressed by genomic mutation, also known as non-functionalization^2^; 2) neo-functionalization occurs when one of the duplicates retains the original function while the other attains a new function that was not present in their ancestral gene^3^,^4^,^5^,^6^; and (3) sub-functionalization is the process in which two duplicated genes retain subsets of their original ancestral function^5^,^7^. During the process of functional divergence, expression divergence is also taking place in the retained copies^8^. The diploidization process eventually retains only a small proportion of duplicated genes, and the majority of redundant copies are inactivated^2^.

However, the questions of short-term gene evolution after WGD and before complete diploidization and of the inactivation rate have not yet been clearly elucidated. WGD is widespread in plant and yeast genomes. The majority of our knowledge regarding genome evolution following WGD comes from plants and yeast. Soybean has undergone two recent WGD events (13 and 59 mya) that have resulted in 75% of its genes being present in multiple copies^9^. Roulin *et al.* found that among recently duplicated genes, only a small proportion (4%) have been either neo- or non-functionalized, but approximately 50% were differentially expressed^9^. These results indicate slow functional divergence but intense expression differentiation. A WGD event was estimated to have occurred in the ancestor of brewer's yeast approximately 100 mya^10^. The descendants of this tetraploid yeast have twice the chromosome number of the pre-WGD yeasts. However, they subsequently deleted approximately 88% of the duplicated genes following WGD^11^. Based on these observations, it is hypothesized that WGDs are followed by massive and rapid gene deletions and intense expression differentiation with slow functional divergence. However, to date, the early evolution of these processes has been poorly investigated in vertebrates because almost all genomes of well-characterised WGDs have returned to a diploid status^12^,^13^.

In comparison with other teleosts, which are widely believed to have gone through three rounds of WGD, common carp (*Cyprinus carpio*) has had an additional round of species-specific genome duplication, called the common carp-specific genome duplication (CcaGD). The common carp chromosome number (n = 50) is twice that of most other teleosts^14^. Previous studies have found more copies of several genes and microsatellites in common carp^15^,^16^. The evidence demonstrates that the genome is still tetraploid and has not been completely diploidized. Various estimates of the timing of the additional round of genome duplication have been made. An analysis of the *c-myc* genes in common carp estimated that the tetraploidization event occurred 58 mya^15^. In another study, Dan *et al.* estimated a tetraploidization time of less than 16 mya^17^. However, with 59 microsatellites, David *et al.* reported that this round of genome duplication occurred approximately 12 mya^16^. Based on a larger dataset of paralogous genes than previous studies, this round of WGD is dated to have occurred 8 mya^18^. The large discrepancy in the estimates of the CcaGD time among previous studies might mainly result from the different number of paralogues examined in each study. Because of the different substitution rates of duplicated gene pairs^18^, estimating substitution rates of a large dataset could provide us a reliable tetraploidization time. These estimates of tetraploidization time demonstrate that the CcaGD is the most recent genome duplication in vertebrates. All of these factors make common carp a rare and unique model to study the early evolution of duplicated genes. In this study, after identifying the pairs of recently duplicated genes from the CcaGD, we found few gene losses, and most pairs were still highly conserved. An expression study revealed massive differentiation that was faster than the functional divergence. Our study provides a comprehensive view of the divergence of expression and function of duplicated genes following recent genome duplication.

## Results

### Identification of recently duplicated genes originating from the CcaGD event

**Figure 1:**
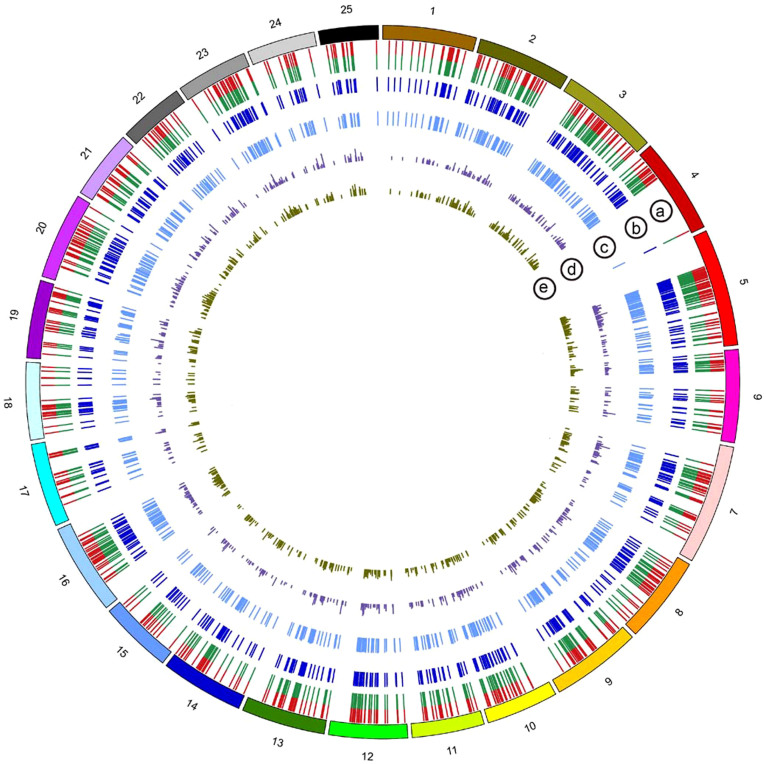
Distribution of recently duplicated common carp genes in the zebrafish genome. The outer circle represents 25 zebrafish chromosomes. (a) The common carp RDG loci in the zebrafish genome. Red and green represent two RDGs. (b) The cDNA identity distribution of RDGs (from 0 to 100%). (c) The protein identity distribution of RDGs (from 0 to 100%). The more similar two copies are, the higher the bar. The cDNA and protein identity distributions demonstrate high conservation between two RDGs. (d) *Ks* of the RDG pairs. (e) *Ka/Ks* of the RDG pairs. The lower bar indicates higher selective pressure.

To analyse gene evolution after WGD in detail, we compared the common carp and zebrafish genomes and obtained 736 pairs of high-confidence double-conserved syntenic (DCS) regions (that is, paralogous regions originating from a WGD, defined in Methods). These DCS regions satisfied a 2:1 mapping ratio with the zebrafish genome. The cross-species mapping of DCS regions is shown in Figure 1. After constructing the DCS regions, we could identify specific recently duplicated genes (RDGs) originating from CcaGD and differentiate them from other duplicated genes from previous rounds of genome duplication.

The WGD event doubled the number of chromosomes and genes. However, subsequent genomic mutation or deletion might result in gene loss, leading to singletons retained in one duplicated scaffold. These 736 DCS regions contain 3,662 genes, which are the descendants of 1,905 pre-CcaGD ancestral genes that are now represented by 148 single-copy genes (singletons) and 1,757 pairs of paralogues (Supplementary Table S1 online). This would mean that only 7.8% of the CcaGD duplicated gene pairs have undergone gene loss and returned to a single-copy state, whereas 92.2% have retained both paralogues. To analyse the early evolution of expression and functional differentiation, we selected these 1,757 paralogous pairs for further study.

### RDG evolutionary rates

**Figure 2:**
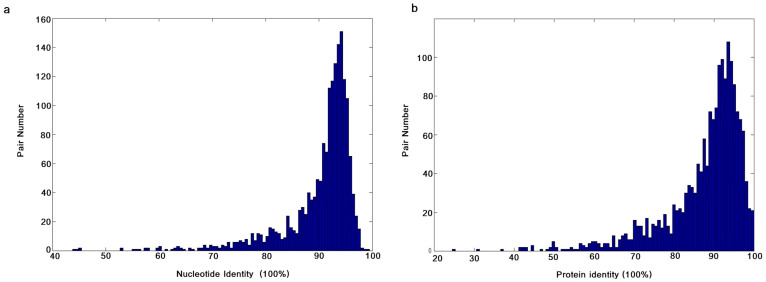
Comparison of RDG sequence identities. (a) cDNA identities of RDG pairs. (b) Protein identities of RDG pairs.

We defined pairs of common carp-specific paralogues identified in DCS regions as RDGs. They were most likely formed by the CcaGD event (Supplementary Fig. S1 online). We were interested in seeing the identities in both nucleotide and protein sequences based on the global sequence alignment between two copies. We found that the RDGs were highly conserved in both nucleotide and amino-acid sequences with two average identities over 90% (Figure 2a and b).

**Figure 3:**
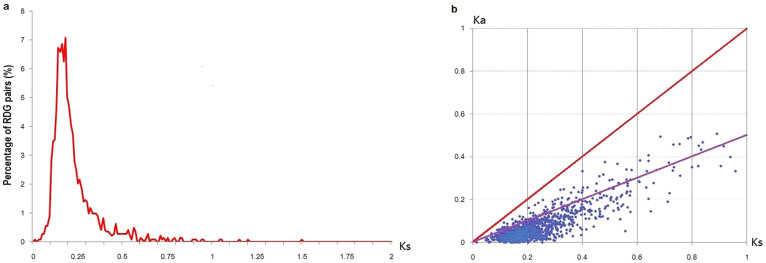
*Ks* and *Ka/Ks* distributions. (a) Histogram showing *Ks* values for RDG pairs. (b) Distribution of *Ka* and *Ks* values. RDG pairs with *Ka/Ks* ratios between 0.5 and 1 are distributed between the red and purple lines.

The *Ka/Ks* ratio was used to test for negative and positive selection^19^. The mean values of *Ks* and the *Ka/Ks* ratios of all RDGs were 0.232 (Figure 3a) and 0.272 (Figure 3b), respectively. We did not observe pairs with *Ka/Ks* ratios greater than 1 (Supplementary Table S2 online). In our study, 121 (6.9%) pairs of paralogues had a *Ka/Ks* ratio between 0.5 and 1, indicating that they were under relaxed purifying selection. Gene Ontology (GO) analysis revealed that these pairs were clearly associated with specific functions using all RDGs as the background (Table 1 and Supplementary Table S3 online). They were enriched in transcription factor activity but significantly rare in transducer activity (corrected P values ≤ 0.05).

### RDG expression patterns

Expression divergence between duplicate genes is always a subject of great interest because it is an essential driving force for the emergence of a new gene originating from a duplication event. The expression data showed that only 48 RDGs had no expressional evidence, supporting the conclusion that most of the duplicate genes were still active (Supplementary Table S4 online).

First, the spatial expression pattern analysis revealed that RDGs in only 131 pairs were not co-expressed because either at least one gene had no expression (FPKM value less than the threshold of active genes, see Methods) or they were active in different tissues. These pairs were considered to have divergent spatial expression. Two copies in 1,332 pairs were co-expressed in at least three tissues (Table 2). Moreover, 972 pairs exhibited co-expression in all six tissues. A previous study in humans reported that duplicated genes exhibited divergent spatial expression^8^. A study in yeast paralogues also revealed rapid divergence in temporal expression between duplicate genes^20^. Our observation differs from these conclusions. Interestingly, we observed that, in 392 pairs, one copy always had higher expression than the others across all six tissues, suggesting the appearance of a dominant copy in the modern genome. To determine whether the proportion of observed 392 pairs was significantly different from what would be expected to occur by chance, we performed a binomial test with the success probability (higher expression across all six tissues) of 0.5. The significant difference (one-tail P value = 8.93 × 10^−10^) from expectation was possibly caused by genetic drift.

Second, expression correlation analysis gave hints regarding whether duplicated genes exhibited transcriptional divergence. Considering the high conservation between two duplicated genes, it is reasonable that two duplicated genes had similar *cis-*regulatory motifs and strongly correlated expression shortly after WGD. Based on this logic, we assumed that two duplicated copies had perfect expression correlation in the early stage and applied permutation test to determine the proportion of duplicated genes that ceased to show strong correlation. We found that 617 pairs (46.32%) had ceased to be strongly correlated (Supplementary Table S5 online).

Third, we performed differential expression analysis on 1,626 pairs of which two copies were co-expressed in at least one tissue. The analysis revealed that 648 of 1,626 pairs (39.9%) were significantly differentially expressed in at least one tissue (Supplementary Table S6 online). GO terms were used to classify genes according to the functions and processes in which they were involved. We found that the terms ‘structural constituent of ribosome', ‘ribosome biogenesis', and ‘ribonucleoprotein complex biogenesis'' contained a significant number of differentially expressed genes compared with the background (Table 3).

### RDG functional divergence

Of the 1,757 RDG pairs, 3,376 genes had domain annotations assigned with Interproscan (Table 4 and Supplementary Table S7 online). There were only 57 pairs in which neither paralogue had a functional domain. The primary reason for this is that they might not have been fully studied because their zebrafish orthologues (44 out of 57 zebrafish orthologues) had no domain information either (Supplementary Table S7 online).

**Figure 4:**
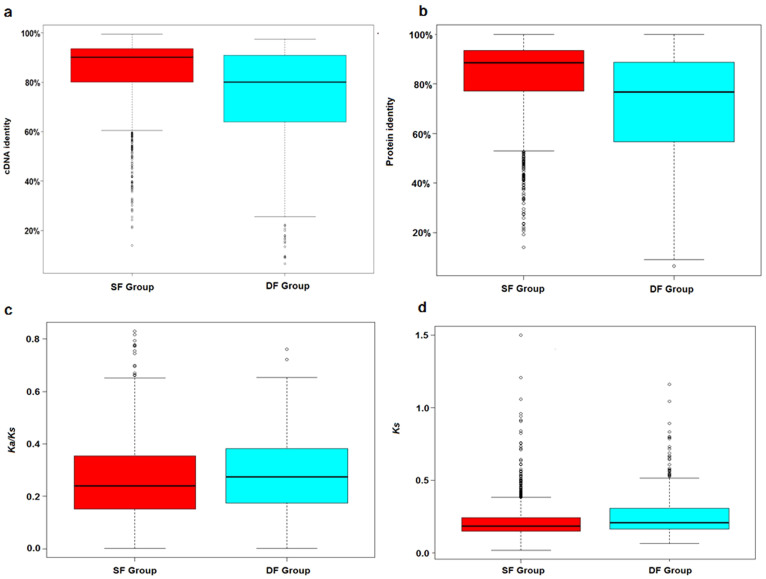
Whisker plots of cDNA and protein identity, *Ks*, and *Ka/Ks* in the SF and DF groups. Whisker plots (with whiskers representing the range of the distribution) showing the sequence conservation for each group of RDG pairs (numbers of RDGs pairs SF = 1,276; DF = 424). The cDNA identities (a), protein identities (b), *Ka/Ks* ratios (c), and *Ks* values (d) were significantly different between the SF and DF groups (Mann–Whitney U test, P values = 8.64 × 10^−28^, 1.24 × 10^−30^, 3 × 10^−3^, and 2.1 × 10^−6^, respectively).

Of the remaining 1,700 pairs, of which at least one copy had domain annotations, two copies in 1,276 pairs had the same functional domains (same function, SF group). This suggests that their functions did not diverge after WGD. The other 424 pairs, of which one copy had a different function from its sister gene, were attributed to the differential function group (DF group), indicating functional divergence in 25% of the pairs (424 out of 1,700). The duplicated pairs in the DF group presented a significantly lower percentage of identity and higher *Ks* and *Ka/Ks* ratios compared with the SF group (Mann–Whitney U test, all P values <0.01), indicating that functional divergence is associated with lower selective pressure on the coding sequences (Figure 4a–d). Furthermore, we examined whether functional divergence was associated with specific classes. GO analysis revealed that the DF group was significantly enriched in multiple functions, including binding, catalytic activity, and immune system processes, whereas the SF group was significantly rare in the above functions (Table 5).

**Figure 5:**
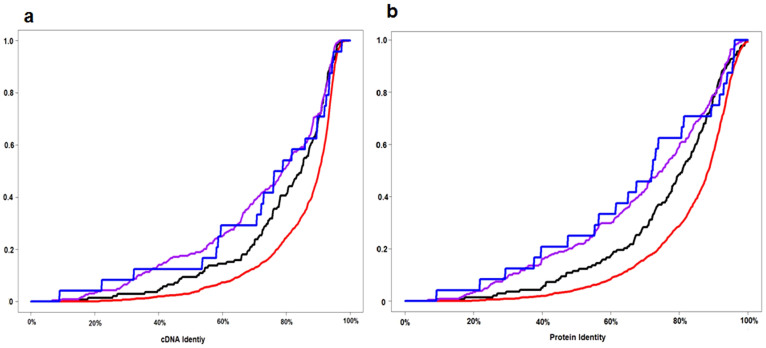
Cumulative distribution curves of cDNA and protein identity in the SF group, Sub-F pair, Non-F pair, and Neo-F pair. Purple: neo-functionalization pair. Blue: non-functionalization pair. Black: sub-functionalization pair. Red: same-function group. (a) cDNA identity among four classes of RDGs. Significant difference among four classes in cDNA identity was proved with the Kruskal–Wallis H test (P value = 6.52 × 10^−22^). Non-F and neo-F pairs did not differ significantly in cDNA identity (Mann–Whitney U test, P value = 0.737), but both had significantly higher substitutions than the sub-F pair (P value = 0.03). The cDNA identity was significantly higher in the SF group than in the sub-F pair (P value = 1.14 × 10^−7^). (b) Protein identities in four classes of RDGs. The Kruskal–Wallis H test (P value = 4.27 × 10^−25^) revealed significant differences among the four classes. Further pair-wise Mann–Whitney U tests indicate that the non-F and neo-F pairs were not significantly different in protein identity (P value = 0.823), but both had significantly higher protein changes than that of the sub-F pair (P value = 0.01). The protein identity was significantly lower in the sub-F pairs than in the SF group (P value = 5.46 × 10^−8^).

To understand the effects of sub-, neo-, and non-functionalization events on the short-term evolution of duplicated genes, we examined whether such events had occurred in the DF group consisting of 424 pairs. In 24 pairs, only one copy had domain annotations, indicating that this gene might have been non-functionalized (Non-F pair). For the other pairs, we compared the copy-specific domains to that of zebrafish orthologues to differentiate neo- and sub-functionalization. In 138 pairs, all copy-specific domains were observed in zebrafish orthologues, suggesting that these pairs were under sub-functionalization (Sub-F pair). In another 228 pairs, no copy-specific domains were found in the zebrafish orthologues, indicating neo-functionalization (Neo-F pair). In the remaining 34 pairs, some copy-specific domains were observed in zebrafish orthologues and some were not, indicating the existence of neo- and sub-functionalization in these pairs. The results revealed that neo- and sub-functionalization were the main outcomes of functional divergence. Comparison of sequence identities among the SF group, Sub-F pairs, Non-F pairs, and Neo-F pairs showed significantly lower conservation in the non- and neo-functionalization pairs than in the SF group and the Sub-F pairs (Figure 5a and b). The results revealed accelerated genomic substitutions in non- and neo-functionalization, moderately rapid substitutions in sub-functionalization, and slow substitutions in function retention.

## Discussion

After WGDs, duplicate genes are subject to the subsequent divergence processes including gene loss, functionalization and expression divergence. The early stages of these processes have not been fully studied at the whole-genome level in vertebrates because, to date, the WGDs in most vertebrates are too ancient to permit such analysis. The common carp WGD is the most recent, and its genome has not yet been completely diploidized. Therefore, it provides a unique opportunity to build a possible scenario for these early stages of gene evolution following a WGD event. Here, we performed a comparative analysis on the fate of recently duplicated genes.

Gene loss, expression differentiation, and protein functionalization are the main driving forces for diploidization. We examined the three possible fates of duplicated genes following WGD. By comparing common carp genes with their zebrafish orthologues, we found that only 7.8% of genes were lost. In most regions, there remains a mapping ratio of 2:1 between common carp and zebrafish genes. Based on studies from plant polyploids and yeast, it has been hypothesized that rapid and intense gene deletions occur after WGDs^21^. However, our results reveal that gene loss was relatively slow after the CcaGD. By contrast, expression divergence and functional differentiation were faster than gene loss. We estimated that the expression of nearly half pairs had ceased to be strongly correlated and that 25% of pairs had functional divergence.

This fast and extensive expression divergence and functionalization might be mainly driven by post-WGD divergence processes. Other biological or experimental mechanisms might also cause expression divergence and functionalization. First, allotetraploidization had effects on gene expression changes. In plants, this event caused genomic changes after hybridization, including part of the expression changes occurring immediately after the WGD^22^. About 5–6% genes had expression alterations in Arabidopsis synthetic allotetraploids compared with their parents^23^, suggesting few but immediate effects on expression changes caused by allotetraploidization. The CcaGD is proposed to be the result of an allotetraploidization^24^. It was possible that some of the expression differences observed in common carp were resulted from allotetraploidization. Secondly, erroneous gene prediction (for instance, missing exons) could result in different functions between duplicated genes or function loss in one copy. Nevertheless, comparing the selected RDGs with the genes of other finished fish genomes revealed almost the same structures among them, demonstrating the high quality of the selected genes (Supplementary Fig. S2 online). The comparison indicated the low probability of erroneous gene prediction. Thirdly, even with correct gene models, accurate annotation of protein function by prediction is a major challenge in the fields of computational and molecular biology. With its laboriousness and expense, experimental characterization of function lags far behind the gene identifications. Therefore, genome-scale gene functions can only be annotated computationally. In this study, the functions of RDGs were predicted with Blast2GO, which reaches an annotation accuracy of 70%^25^. In terms of the accuracy, Blast2GO is superior to other methods which are based on homology search^26^, indicating that this method performs well enough to guide experiments and functions comparison.

It is interesting to investigate the correlation between protein divergence and expression divergence. A number of studies have investigated the relationship between coding sequence divergence and expression divergence. Makova and Li found a significant negative correlation between expression correlation and both *Ks* and *Ka* in human duplicate gene pairs for *Ka* < 0.2^27^. For all pairs, we carried out the correlation between *Ks* (or *Ka*) and expression correlation. No significant correlation was found between the expression correlation coefficient and *Ks* (*R* = −0.029, Student's *t-*test P value = 0.34) or between the expression correlation coefficient and *Ka* (*R* = −0.024, Student's *t-*test P value = 0.43) (Supplementary Table S8 online). Further detailed analysis on pairs under different ranges of *Ks* (or *Ka*) confirmed no significant relationship between the expression correlation coefficient and *Ks* (or *Ka*) (Supplementary Table S9 and S10 online). These results suggested that expression dissociation and function specification were two independent processes and that non-coding (especially *cis-*regulatory) sequence changes and coding divergence were decoupled. The inconsistency between our analysis and previous studies might be because, to date, WGDs studied in other species are more ancient than that of common carp.

The expression analysis revealed that most RDGs had transcription signals and supported our observation that few genes were lost in a short time following WGD. The analysis also showed the co-expression of most duplicated genes with few pairs exhibiting divergent spatial expression. This result is quite different from observations in human and other species in which duplicated genes became specialized in their expression patterns with decreased breadth and increased specificity of expression^27^. The difference is mainly because WGDs in these species are extremely ancient, and the expression of most duplicated genes has diverged. Despite co-expression, we observed that nearly half pairs ceased to be strongly correlated and that 39.9% of pairs were differentially expressed. These results suggested slow spatial divergence but rapid expression dissociation. Considering that specific expression is the terminal fate of the copies after the diploidization process, it is therefore possible that the expressions of all duplicated copies first ceased to be correlated, despite being co-expressed in many tissues, which was followed by divergence in spatial expression at the end of the diploidization process.

We investigated the proportions of the non-, neo-, and sub-functionalized pairs in all RDGs. A few pairs (57 out 1,757) might have experienced non-functionalization. Although 75% of RDGs retained the same functions as their sister copies, function specification occurred in the other 424 pairs. Neo- and sub-functionalization occurred in most of the functionalized pairs, suggesting that they were the main driving force behind functionalization. Neo- and sub-functionalization can be achieved through amino acid changes^8^. Indeed, we found accelerated genomic substitutions in neo-functionalization and moderately rapid substitutions in sub-functionalization pairs.

Common carp has undergone a recent round of whole genome duplication, making it an ideal model with which to investigate gene evolution following WGD. We studied the sequence identities, evolutionary rates, functional divergence, and expression patterns of recently duplicated genes. We found that most sister copies were retained, suggesting that gene deletion was a slow process and is still in progress in the common carp genome. A large proportion of recently duplicated genes exhibited expression differentiation and/or functional divergence. Altogether, analysis of the common carp genome revealed that of the three potential fates, expression dissociation and protein functionalization dominated and were fast in comparison to gene loss, challenging the current hypothesis of extensive gene deletions following WGD.

## Methods

### Data sources

The European common carp genome was downloaded from ZFgenomics^28^. Previously, we generated transcriptome sequencing across six common carp tissues (brain, skin, gill, blood, head kidney, and muscle) on the Illumina HiSeq 2000 platform (Illumina, San Diego, USA)^29^. The Illumina RNA-seq reads were aligned to the genome for gene prediction and expression analysis.

### Identification of duplicated syntenic regions and recently duplicated genes

We predicted consensus gene models by combining *ab initio* prediction, homologous gene prediction, and RNA-seq models. Briefly, Fgenesh^30^ was used for *ab initio* gene prediction. Zebrafish, tetraodon, fugu, medaka, stickleback, and human proteins from the Ensembl database^31^ were aligned to the genome using BLAT^32^ with an identity cutoff of 70%. RNA-seq reads were aligned to the genome using TopHat, and RNA-seq gene models were built using Cufflinks^33^. Three sets of gene models were merged into a consensus gene set with Cuffmerge in the Cufflinks package. Sequence conservation can help eliminate false positives^34^. The false positives resulted from the low specificity of the *ab initio* gene prediction software^35^ and non-coding genes in RNA-seq models^36^. We retained those consensus gene models with homologous genes as evidence for further analysis.

To investigate the existence of a species-specific WGD and identify the recently duplicated genes (RDGs), we identified double-conserved syntenic (DCS) regions (that is, paralogous regions originating from the CcaGD) based on the closely matching orthologues between common carp and zebrafish following Kellis *et al.*'s method^37^. A closely related species that descended from a common ancestor and diverged from the study species before duplication was selected as the reference. Zebrafish and common carp belong to the *Cyprinidae* family. After separation, common carp had one more round of duplication than zebrafish. The close relationship between common carp and zebrafish offers an opportunity to identify RDGs from the CcaGD and study their early evolution.

A DCS block is defined as a series of genes in the non-duplicated species that is found on two different chromosomes in another species that underwent an additional WGD^13^. We identified DCS blocks on the basis of the orthologous relationship between common carp and zebrafish. First, we aligned common carp genes against zebrafish genes using BLASTP with an e-value of 10^−5^. For each common carp gene, we selected the most similar zebrafish gene as its orthologue. Second, we identified conserved syntenic scaffolds to zebrafish genome. Considering that speciation between common carp and zebrafish took place ~120 mya^18^, genomic rearrangement might have taken place. Therefore, a common carp DCS block syntenic to a zebrafish chromosome might include genes orthologous to another zebrafish chromosome. The scaffolds within at least three genes and over half of the genes orthologous to one zebrafish chromosome were considered to be conserved syntenic regions. Finally, we searched for the duplicated syntenic scaffolds homologous to the same zebrafish chromosomal region. If two conserved syntenic common carp scaffolds were homologous to the same chromosomal region in zebrafish, they were considered to be double-conserved syntenic scaffolds originating from the CcaGD. DCS blocks can be very short because they are dependent on assembly continuity and scaffold anchoring. Pairs of paralogous carp genes on two different scaffolds that belong to a DCS block are most likely duplicates originating from the CcaGD event and are called RDGs. In a DCS block, genes syntenic to zebrafish but without paralogous genes on the other scaffold are most likely former CcaGD duplicates in which one of the duplicated genes was lost. These are referred to as singletons. For further functional comparison, we predicted the Gene Ontology information for RDGs by Blast2GO^26^.

### Global sequence alignment and *Ka/Ks* calculation

Having identified RDGs, we estimated nucleotide and amino acid identities to detect cases of accelerated evolution. We used Needle in the EMBOSS package^38^ to construct global protein and nucleotide alignments and identify their sequence similarity.

For each pair, the number of non-synonymous substitutions per non-synonymous site (*Ka*) to the number of synonymous substitutions per synonymous site (*Ks*), and the *Ka/Ks* ratio were calculated using PAML^19^, based on the alignment of both the nucleotide and protein sequences. To minimize the statistical artefacts that can arise because of a saturation of *K*s, we discarded the pairs that had *Ks* > 2^39^. The *K*s frequency in each interval size of 0.01 within the range [0–2.0] was plotted.

### Gene Ontology (GO) enrichment analysis

To investigate the biological consequence of expressional differentiation or functional divergence, we used WEGO^40^ to identify significantly enriched molecular functions and biological processes in one specific group of RDGs, using all identified RDGs as the background. WEGO uses the Pearson Chi-Square test to indicate significant relationships between two input datasets. Then, the P value was corrected for multiple testing with the Benjamini-Hochberg method^41^. The molecular functions and biological processes with corrected P values ≤ 0.05 were considered to be statistically enriched in this group.

### RDG expression patterns

Illumina RNA-seq reads of six tissues were aligned to the common carp genome using TopHat^42^. Considering that the high sequence similarity of duplicated genes might lead to the multiple alignment of sequencing reads, read counts used in expression analysis were based on a subset of uniquely aligned reads, following the strategy of Roulin *et al.*^9^. That is, we discarded the reads that were mapped to both gene copies. For a given gene, the expression in each sample was counted with reads that were uniquely mapped to its region and normalized by its length in Cufflinks^33^. FPKM (Fragments Per Kilobase of transcript per Million fragments) was applied to measure the normalized expression value. Studies suggested that the low-abundance transcripts were likely the by-products of biological or experimental noise rather than active genes involved in the biological processes^43^,^44^. Hart *et al.* provided a robust FPKM threshold of 0.213 (log_2_FPKM of -2.23) to differentiate active gene expression from background noise^44^. Herein, for a given gene, if the expression level in one tissue was over the threshold, it was considered as active. Otherwise, its expression was likely from background noise.

We employed three different measures of the expression divergence between two duplicated genes. First, we studied the dynamics of spatial expression divergence in RDGs. A study of temporal expression in yeast paralogues revealed rapid divergence in temporal expression between duplicate genes^20^. We focused on the question of whether the study of spatial expression of common carp RDGs was consistent with that conclusion. If the FPKM values of both copies were over the threshold of active genes in a given tissue, they were considered as ‘co-expressed'. Or if only one copy had an FPKM value over the threshold, then two duplicate genes did not co-express in this tissue. Two copies without co-expression in all six tissues were said to have divergent spatial expression.

Second, we investigated the expression correlation of two copies. After WGD, two duplicated genes are initially likely to have identical *cis-*regulatory motifs. Therefore, it is logical that the expression of two copies are strongly correlated in the early stage^45^. Using this logic, we assumed that the theoretical Pearson correlation coefficient (*R*) of two duplicated genes was 1 in the early stage of WGD and investigated how many pairs had departed from perfect correlation. We calculated the expression *R* between two copies over the tissues studied. To investigate whether the *R* of one pair was statistically less than 1, we determined the empirical threshold at the one-tailed level of 0.05 with permutation test. For two copies in every tissue where they were co-expressed, all reads that were mapped to them, were randomly assigned to each copy. Then the *R* was computed based on randomly drawn reads. The above two steps were repeated 1000 times. An empirical threshold at the one-tailed 0.05 level was determined based on the 1000 simulated *R* values. If the actual *R* was less than the threshold, the actual *R* significantly differed from the theoretical *R* and this pair was considered to have correlation divergence. Otherwise, this pair had strongly correlated expression as the early stage.

Third, in each tissue where two RDGs were co-expressed, differential expression was tested for two RDGs using DEGseq^46^. P values were adjusted to maintain the false discovery rate (FDR) at 0.05 using the Benjamini–Hochberg method^41^. The differentially expressed gene pairs were obtained with the FDR-corrected P value cutoff of 0.05.

### Comparing the functions of RDGs

We assigned functional domains for RDGs using Interproscan^47^, which scanned protein domains and important sites to determine potential protein functions. To detect whether functional divergence existed after a WGD, we compared the domains of two copies. Those pairs in which two copies had the same domains were attributed to the same function group (SF group); the other pairs in which two copies had distinct domains belonged to the differential function group (DF group). Accumulated genomic substitutions eventually lead to functional divergence^48^. Thus, we examined whether the sequence similarities and evolutionary rates were different between the SF and DF groups.

Additionally, we investigated the existence and frequency of non-, sub-, and neo-functionalization events in the DF group by comparing the annotations of two copies. If one partner had functional domains or important sites while the other did not, we then concluded that a non-functionalization event had occurred in this pair. For those pairs in which two copies had domains, if one copy had different domains from the other, it might have resulted from either neo- or sub-functionalization. To differentiate between neo- and sub-functionalization, we compared the RDG domains with their zebrafish orthologues. We downloaded the zebrafish orthologue domains from Ensembl database^31^. If the copy-specific domains were observed in zebrafish orthologue, the divergence might have resulted from sub-functionalization. Otherwise, the divergence might have been caused by neo-functionalization. Mutations resulted in functionalization and decreased similarity between two paralogues. We investigated the sequence similarity level in pairs of non-, neo-, and sub-functionalization.

## Author Contributions

J.T.L. and X.W.S. have contributed to the conception and design of the study, and the critical revision of the article. J.T.L., G.Y.H. and X.F.K. analyzed the data. H.D.L. and J.T.L. performed permutation test. G.Y.H. and J.M.Z. prepared figures. J.T.L. drafted the article. C.Y.L., G.B.X., and X.M.L. participated in the acquisition of data and statistical analysis. All authors reviewed the manuscript.

## Supplementary Material

Supplementary Information supplementary information

Supplementary Information Table S1

Supplementary Information Table S2

Supplementary Information Table S3

Supplementary Information Table S4

Supplementary Information Table S5

Supplementary Information Table S6

Supplementary Information Table S7

Supplementary Information Table S8

## Figures and Tables

**Table 1:** GO enrichment analysis of RDGs under relaxed purifying selection

GO Term	GO Description	Proportion*	Corrected P value
GO:0003700	sequence-specific DNA binding transcription factor activity	13.3:5.4&	0
GO:0030528	transcription regulator activity	15.2:6.6&	0
GO:0005515	protein binding	33.6:45.4#	0.023
GO:0007154	cell communication	13.3:22.6#	0.023
GO:0004871	signal transducer activity	2.4:8.4#	0.035
GO:0060089	molecular transducer activity	2.4:8.4#	0.035

*Proportion: the first number is the proportion of RDGs under relaxed purifying selection, and the second number is the percentage of all RDGs.

^#^Functions or processes in which RDGs under relaxed purifying selection were significantly rare.

^&^Functions or processes in which RDG pairs under relaxed purifying selection were enriched.

**Table 2:** Spatial expression analysis of RDGs across six tissues

Type		Number of RDG pairs
Divergent spatial expression		131
Co-expression in at least one tissue	Co-expression in one tissue	171
	Co-expression in two tissues	123
	Co-expression in three tissues	97
	Co-expression in four tissues	93
	Co-expression in five tissues	170
	Co-expression in six tissues	972
Total		1,757

**Table 3:** Enriched GO functions and processes of the differentially expressed gene pairs

GO term	GO Description	Proportion*	Corrected P value
GO:0003735	Structural constituent of ribosome	2.5:1.1	0.038
GO:0042254	ribosome biogenesis	3.4:1.8	0.038
GO:0022613	Ribonucleoprotein complex biogenesis	4.0:2.2	0.038

*Proportion: the first number is the associated gene number to this term among all differential expressed genes, and the second number is the percentage of the associated gene number out of all RDGs.

**Table 4:** Classification of RDG pairs based on domain annotation

Type		Number of RDG pairs
Both copies without domain information		57
Group of same function	Domains of both copies were the same	1,276
Group of differential function	Only one copy had no domain information	24
	Both copies had domain information but only one had different domains from the other copy	400
Total		1,757

**Table 5:** Enriched GO functions and processes of the DF group

GO Term	GO Description	Proportion*	Corrected P value
GO:0000166	Nucleotide binding	25.9:16.7	0
GO:0001882	Nucleoside binding	15.6:9.9	0
GO:0002376	Immune system process	8.4:5.2	0
GO:0002682	Regulation of immune system process	3.8:1.6	0
GO:0003824	Catalytic activity	44.4:35.4	0
GO:0005488	Binding	81.7:71.6	0
GO:0016491	Oxidoreductase activity	7.3:4.3	0
GO:0031294	Lymphocyte costimulation	3.8:1.6	0
GO:0044237	Cellular metabolic process	53.8:47.0	0
GO:0005515	Protein binding	51.6:45.4	0.02
GO:0055114	Oxidation-reduction process	6.1:3.6	0.02

*Proportion: the first number is the associated gene number to one term among all genes in the DF group, and the second number is the percentage of the associated gene number among all RDGs.
