# Supplementary material for: The fate of recent duplicated genes following a fourth-round whole genome duplication in a tetraploid fish, common carp (Cyprinus carpio)
Source: Sci Rep. 2015 Feb 3;5:8199. doi: 10.1038/srep08199 (PMC4314655; doi:10.1038/srep08199)
Supplement: Supplementary Information [file srep08199-s1.pdf]

SUPPLEMENTARY INFORMATION FOR

The fate of recent duplicated genes following a fourth-round whole genome duplication in a tetraploid fish, common carp (*Cyprinus carpio*)

Jiong-Tang Li[1,†,*] , Guang-Yuan Hou[1, †], Xiang-Fei Kong[1,2], Chun-Yan Li[1,2,3], Jian-Ming Zeng[1], Heng-De Li[1], Gui-Bao Xiao[1,4], Xiao-Min Li[1,4], Xiao-Wen Sun[1]

† Equal contributors

Affiliations

[1] The Centre for Applied Aquatic Genomics, Chinese Academy of Fishery Sciences, Beijing, 100141, China

[2] College of Fisheries and Life Science, Shanghai Ocean University, Shanghai, 201306, China

[3] Tianjin Fisheries Research Institute, Tianjin, 300221, China

[4] College of Life Science and Technology, Dalian Ocean University, Dalian, 116023, China

Correspondence and requests for materials should be addressed to J.T. L. (lijt@cafs.ac.cn)

Supplementary Figures

Figure S1. DCS scaffolds of common carp

Comparison with zebrafish genome (orange) reveals the duplicated nature of the common carp genome (purple and blue). Recent duplicated genes were identified in comparison to zebrafish genes.

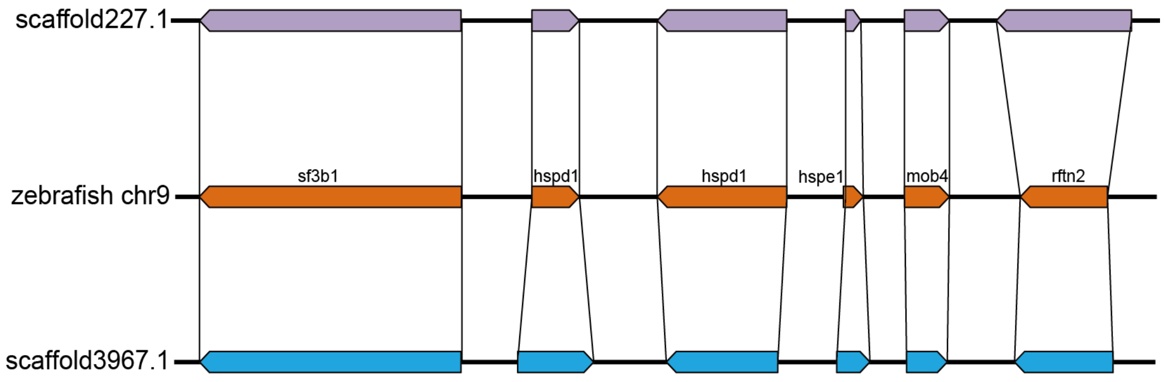

Figure S2. Comparison of common carp RDGs to other teleosts

We compared the proteins of RDGs to the ones of other seven teleost fish. The comparison revealed almost the same length distributions among them, demonstrating the high-quality of the selected genes.

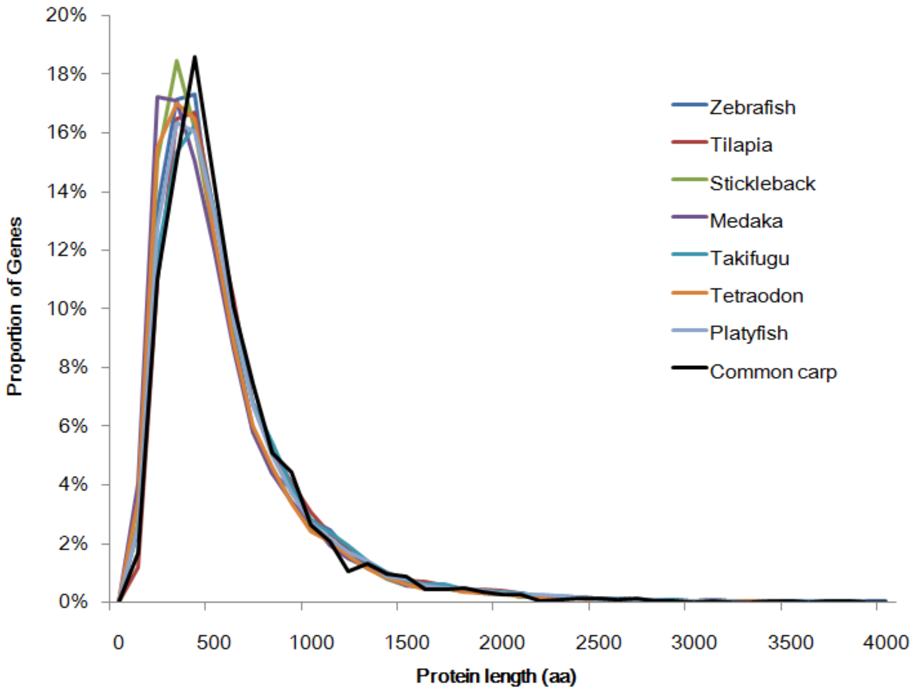

Supplementary Tables

Table S1. Common carp RDGs and zebrafish orthologs.

(Included in a separated excel file)

Table S2. *Ka/Ks*, *Ka* and *Ks* values of RDGs.

(Included in a separated excel file)

Table S3. Gene Ontology annotations of RDGs.

(Included in a separated excel file)

Table S4. Expression values of RDGs across six tissues.

(Included in a separated excel file)

Table S5. Sampling analysis of RDG expression correlation.

(Included in a separated excel file)

Table S6. Differential expression analysis of RDG pairs in each tissue.

(Included in a separated excel file)

Table S7. Domain annotations of RDGs with Interproscan

(Included in a separated excel file)

Table S8. *Ka*, *Ks* and expression correlation coefficient of each pair.

(Included in a separated excel file)

Table S9. The relationship between *Ka* and the expression correlation coefficient across duplicate genes

Ka	Pair number	*R* *	Student *t*-test P value
≥ 0 and < 0.02	192	-0.046	0.524
≥ 0.02 and < 0.04	316	-0.056	0.318
≥ 0.04 and < 0.06	212	-0.046	0.505
≥ 0.06 and < 0.08	103	-0.105	0.291
≥ 0.08 and < 0.10)	65	0.09	0.478
≥ 0.10 and < 0.12)	43	0.047	0.763
≥ 0.12 and < 0.14)	37	0.231	0.169
≥ 0.14 and < 0.16)	23	0.267	0.218
≥ 0.16 and < 0.18)	20	0.248	0.291

≥ 0.18 and < 0.20)	16	-0.009	0.973
≥ 0.20	65	-0.035	0.782

* Here we calculated the *R* between *Ka* and the expression correlation coefficient.

Table S10. The relationship between *Ks* and the expression correlation coefficient across duplicate genes

Ks	Pair Number	*R* *	Student *t*-test P value
≥ 0 and < 0.05	2		
≥ 0.05 and < 0.1	25	-0.345	0.091
≥ 0.1 and < 0.15	239	-0.048	0.457
≥ 0.15 and < 0.2	340	0.032	0.551
≥ 0.2 and < 0.25	194	0.009	0.905
≥ 0.25 and < 0.3	98	0.139	0.173
≥ 0.3 and < 0.35	66	0.085	0.499
≥ 0.35 and < 0.4	45	-0.174	0.254
≥ 0.4 and < 0.45	17	0.36	0.155
≥ 0.45 and < 0.5	15	0.086	0.761
≥ 0.5	51	-0.073	0.610

* Here we calculated the *R* between *Ks* and the expression correlation coefficient.
